# Ab Initio Study of the Interaction of a Graphene Surface Decorated with a Metal-Doped C_30_ with Carbon Monoxide, Carbon Dioxide, Methane, and Ozone

**DOI:** 10.3390/ijms23094933

**Published:** 2022-04-29

**Authors:** Mónica Canales, Juan Manuel Ramírez-de-Arellano, Juan Salvador Arellano, Luis Fernando Magaña

**Affiliations:** 1 Universidad Autónoma Metropolitana Unidad Azcapotzalco, Av. San Pablo Xalpa No. 180, Colonia Reynosa Tamaulipas, Delegación Azcapotzalco, Ciudad de México 02200, Mexico; monic.canales@gmail.com (M.C.); jsap@azc.uam.mx (J.S.A.); 2 Escuela de Ingeniería y Ciencias, Tecnologico de Monterrey, Av. Eugenio Garza Sada 2501, Monterrey 64849, Mexico; 3 Instituto de Física, Universidad Nacional Autónoma de México, Apartado Postal 20-364, Ciudad de México 01000, Mexico

**Keywords:** carbon nanostructures, semifullerene, pollutant molecules, adsorption, graphene, carbon monoxide, carbon dioxide, methane, ozone

## Abstract

Using DFT simulations, we studied the interaction of a semifullerene C_30_ and a defected graphene layer. We obtained the C_30_ chemisorbs on the surface. We also found the adsorbed C_30_ chemisorbs, Li, Ti, or Pt, on its concave part. Thus, the resulting system (C_30_-graphene) is a graphene layer decorated with a metal-doped C_30_. The adsorption of the molecules depends on the shape of the base of the semifullerene and the dopant metal. The CO molecule adsorbed without dissociation in all cases. When the bottom is a pentagon, the adsorption occurs only with Ti as the dopant. It also adsorbs for a hexagon as the bottom with Pt as the dopant. The carbon dioxide molecule adsorbs in the two cases of base shape but only when lithium is the dopant. The adsorption occurs without dissociation. The ozone molecule adsorbs on both surfaces. When Ti or Pt are dopants, we found that the O_3_ molecule always dissociates into an oxygen molecule and an oxygen atom. When Li is the dopant, the O_3_ molecule adsorbs without dissociation. Methane did not adsorb in any case. Calculating the recovery time at 300 K, we found that the system may be a sensor in several instances.

## 1. Introduction

Molecules, such as CO, CO_2_, CH_4_, and O_3,_ are air and water pollutants that threaten the environment and life, prompting the scientific community to develop technological solutions to such challenges [1,2,3]. In this study, we are interested in exploring the use of fullerenes for such aims.

Surfaces based on fullerenes and their variations have been widely studied since the prediction and further synthesis of the C_60_ structure [4,5,6], a highly stable group of molecules consisting of 60 carbon atoms, also named buckminsterfullerene, buckyball, or simply fullerene. Although fullerenes, such as C_60_, C_70_, or larger, are the most commonly studied [7,8], smaller fullerenes can also be experimentally produced and are of particular interest due to their curvature [9,10,11].

Fullerene fragments such as a C_30_ hydrocarbon—i.e., half of the buckminsterfullerene C_60_—can show some of the properties of their complete counterparts [9] while also offering new possibilities due to their open basket-like shape. Similar nonplanar-related structures are corannulene (C_20_H_10_) and coronene, known since the 1960s [12,13,14]. The latter is a bowl carbon structure with 20 atoms or C_20_, the smallest possible fullerene, which has been experimentally produced [11]. And the discovery of bidimensional, planar structures, such as graphene [15,16] and borophene [17], has also attracted attention because of their attractive properties and potential applications.

Previous investigations from other authors considered fullerenes on a graphene surface, focusing on studying weak interactions at a molecular level [18]. Graphene can accept electrons from a C_60_ fullerene relatively quickly, which, combined with the high transport capability of the former, turns this hybrid material into a good candidate for solar cell technology [19]. The development of hybrid surfaces has also focused on fabricating graphene-C_60_ films on silicon surfaces by a multistep self-assembly process [20]. The potential applications of these systems are promising, especially as lubricating films in electromechanics microsystems. Graphene-C_60_ vertical heterostructures composed of C_60_ thin films have also focused on their structural and electrical properties [21]. The absorption of pollutants, such as COCl_2_ (phosgene), H_2_S, CO, or CO_2_, among others, by these hybrid structures has also raised attention. Decorating such arrangements with transition metals usually catalyzes absorption [22,23,24,25].

This work studies a mixed surface formed by a semifullerene C_30_ adsorbed on a defective 5 × 5 graphene layer without a hexagonal ring, i.e., six carbon vacancies. The roughness of the surface at several sites and the change in curvature make this an attractive system to dope with different atoms. We considered Li, Ti, and Pt-decorations and then studied the ability of the compound system to capture the pollutant molecules mentioned above. We found that all the molecules reacted with the surface except methane.

## 2. Results

### 2.1. Optimization of the Semifullerene C_30_

**Figure 1:**
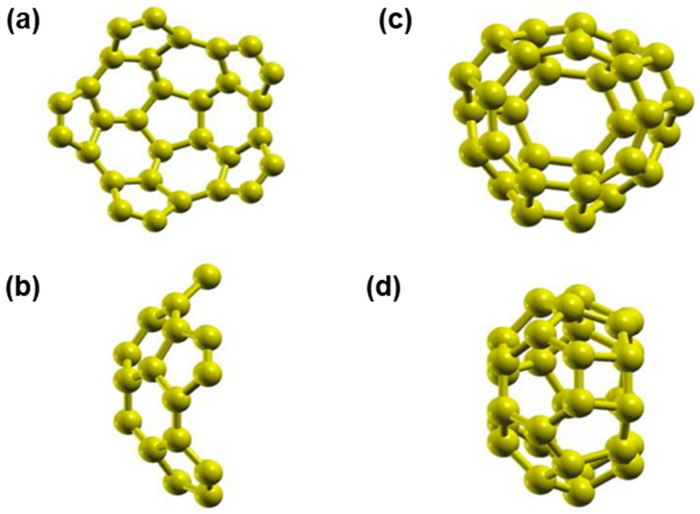
Molecules after optimization of C_30_. In (**a**,**b**), we have C30 with a pentagonal base, in a front and a side view, respectively. In (**c**,**d**), we show a front and side view for C_30_ with a hexagonal base.

We took two different parts when splitting a fullerene C_60_ into two halves (“buckyballs”) [10] to obtain a semifullerene C_30_. One has a pentagon in the base (section P), and the other has a hexagonal base (section H). Figure 1 shows the optimization for each case. Figure 1a,b show the C_30_ with a pentagon at the bottom, and Figure 1c,d show the C_30_ with a hexagonal base. After optimization, we discovered that in the C_60_ molecule, the separation between the carbon atoms is 1.425 Å. For section P, the distance is 1.444 Å at the bottom, and for the rest of the particles, the average separation is 1.375 Å. For section H, the space is 1.485 Å at the base, and the average spacing is 1.436 Å for the other particles. The results from other authors [10] agree with our results.

### 2.2. Optimization of Graphene with a Six-Vacancy Cluster

**Figure 2:**
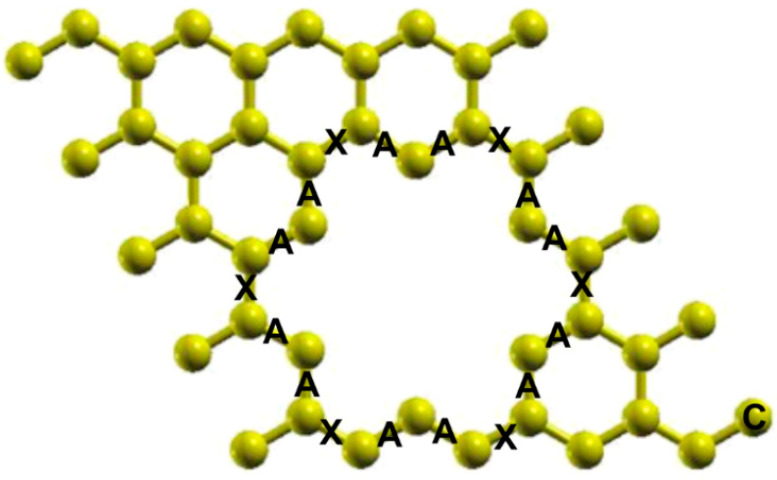
Unit cell after optimization of graphene with a six-vacancy cluster. The bond lengths marked with A are 1.403 Å, and those marked with X are 1.452 Å. The other bonds are the same size as in pristine graphene, 1.420 Å.

The vacancies in the graphene layer are necessary for the adsorption of the C30 molecule. We considered a graphene unit cell with 50 atoms and made a six vacancy cluster. Then, we optimized the system. Figure 2 shows the final configuration. We note that there is some distortion in the graphene lattice. The carbon atoms around the vacancies have different separations concerning pristine graphene. The bond lengths marked with A are 1.403 Å, and those marked with X are 1.452 Å. The other bonds are 1.420 Å, which is the same size as pristine graphene.

### 2.3. Adsorption of the C_30_ Molecule with a Pentagonal Base

**Figure 3:**
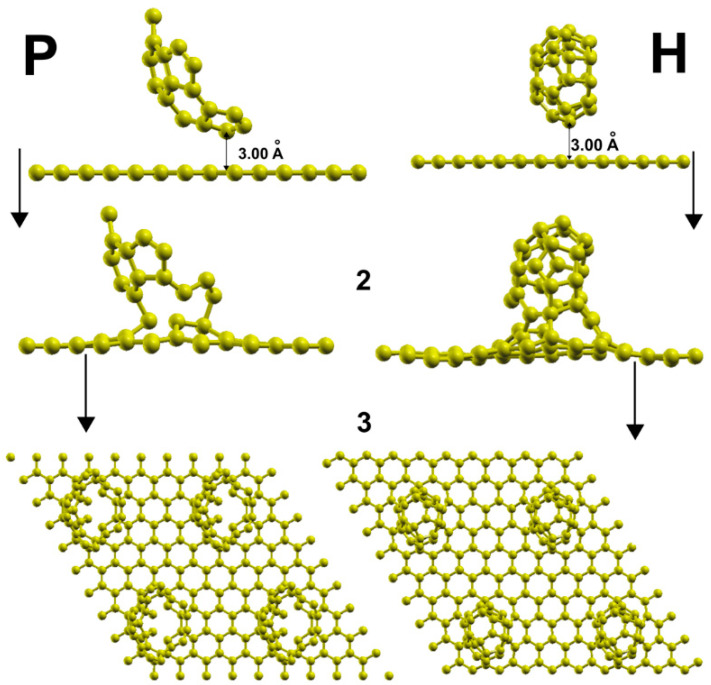
Adsorption of C_30_ on graphene with a six-vacancy cluster. In column P, we show the adsorption of the C_30_ molecule with a pentagonal base. In the same column P, in row 1, we have the initial location of the semifullerene. We have the final configuration after adsorption in the second row of the same column. We view the surface with four-unit cells from above in the last row of this column, P. The corresponding sequence for a C_30_ with a hexagonal base is in column H.

The left column (P) in Figure 3 shows the adsorption of the C_30_ molecule with a pentagonal base in row 1. The initial location of the C_30_ molecule is above the cluster vacancies. Besides, the molecule is, with the closest carbon atom to the surface, at a distance of 3 Å. In the same column, row 2, we can see the system’s final configuration. The adsorption energy is −15.29 eV, indicating a powerful graphene reaction. We perceive a view from above, the graphene surface in row 3 of the same column after adsorption using four-unit cells.

### 2.4. Adsorption of the C_30_ Molecule with a Hexagonal Base

Column H in row 1 shows the initial location of the C_30_ molecule with a hexagonal bottom concerning the graphene layer with the closest carbon atom to the surface at a distance of 3 Å. In the same column, row 2, we can see the system’s final configuration. The adsorption energy is −16.410 eV, which is a stronger adsorption than in the pentagonal case. We perceive the graphene surface in row 3 of the same column after adsorption using four-unit cells.

### 2.5. Adsorption of Metals on the Graphene-C_30_ (P) Surface

#### 2.5.1. Doping with Li

**Figure 4:**
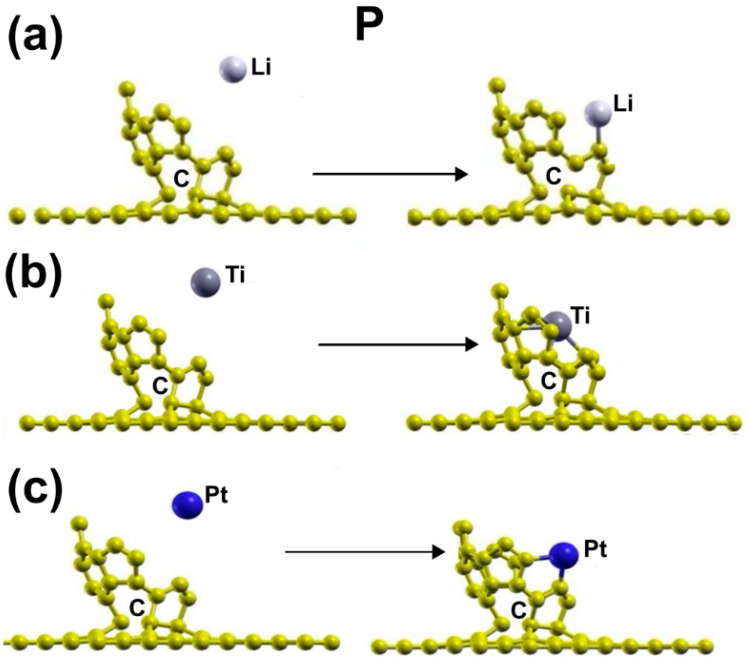
Adsorption of Li, Pt, and Ti on the graphene-C_30_ system for the pentagonal base. The three metals adsorbed with a strong reaction on the surface. (**a**) presents the initial and final configuration for the adsorption of a lithium atom on the surface. (**b**) shows the initial and final configuration for the adsorption of a titanium atom on the surface. (**c**) shows the initial and final configuration for the adsorption of a platinum atom on the surface.

Figure 4a presents the initial and final configuration for the adsorption of a lithium atom on the surface. The initial distance between the metal atom and the plane defined by the opening of C_30_ was 3.27 Å and 5.27 Å from the graphene layer. The lithium atom ends up bound to a carbon atom of the C_30_. The adsorption energy of Li is –3.686 eV, which indicates a strong reaction with the surface. The Li atom yields 0.0561 electrons.

**Figure 5:**
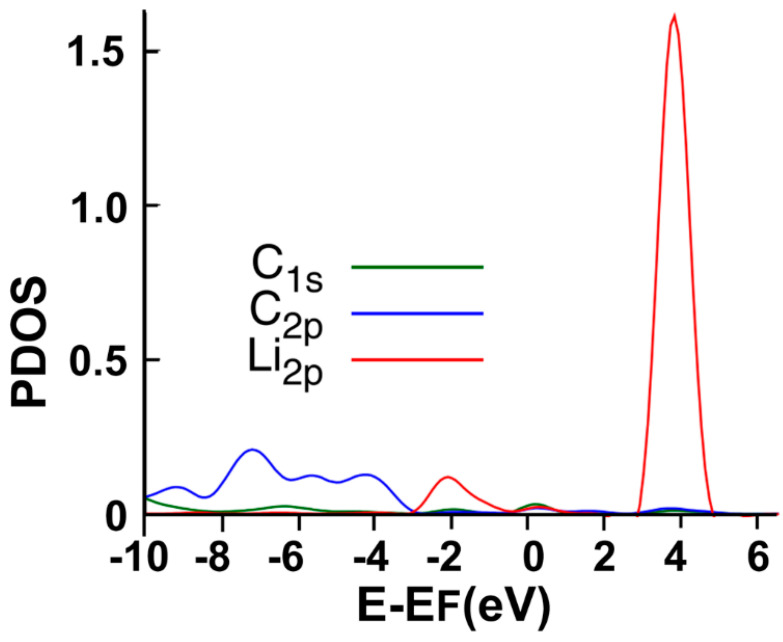
The PDOS for the adsorption of Li on the graphene-C_30_ system for the pentagonal base.

Figure 5 shows the interaction’s projected density of states (PDOS). Note the hybridization of orbitals s and p from carbon with the orbital p from lithium around the Fermi energy at around 4 eV above the Fermi energy and about 2 eV below the Fermi energy.

#### 2.5.2. Doping with Ti

Figure 4b shows the initial and final configuration for the adsorption of a titanium atom on the surface. The initial distance between the metal atom and the plane defined by the opening of C_30_ was 3.34Å and 5.25 Å from the graphene layer. The titanium atom ends up bound to four carbon atoms of the C_30_. The adsorption energy is = −8.082 eV, implying an intense reaction. The Ti atom yields 0.6129 electrons to the surface.

**Figure 6:**
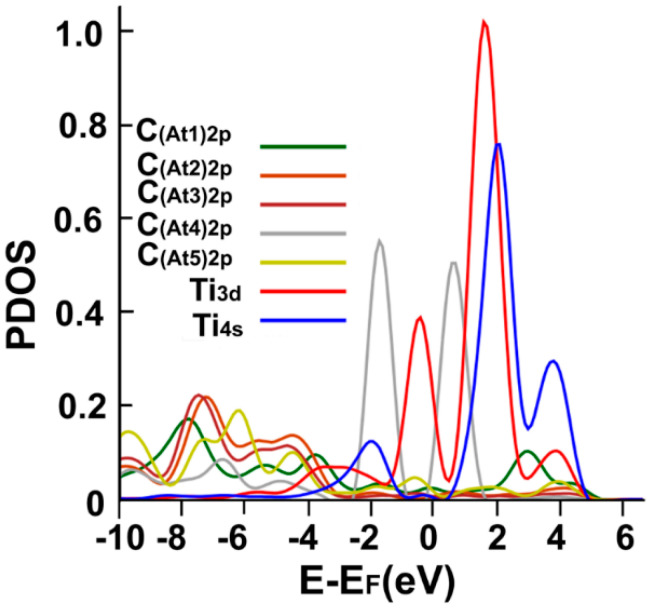
The PDOS for the adsorption of Ti on the graphene-C_30_ system for the pentagonal base.

We can see in Figure 6 the interaction’s PDOS. We note the hybridization of orbitals s and d from titanium with the orbitals p from the neighboring carbon atoms between −4 eV, a bit below the Fermi energy, and between 1 eV and 5 eV.

#### 2.5.3. Doping with Pt

Figure 4c shows the initial and final configuration for the adsorption of a platinum atom on the surface. The initial distance between the metal atom and the plane defined by the opening of C_30_ was 3.36Å and 5.24 Å from the graphene layer. The platinum atom ends up bound to two carbon atoms of the C_30_, with an adsorption energy of −5.982 eV, showing a strong reaction with the surface again. The Pt atom yields 0.3910 electrons to the surface.

**Figure 7:**
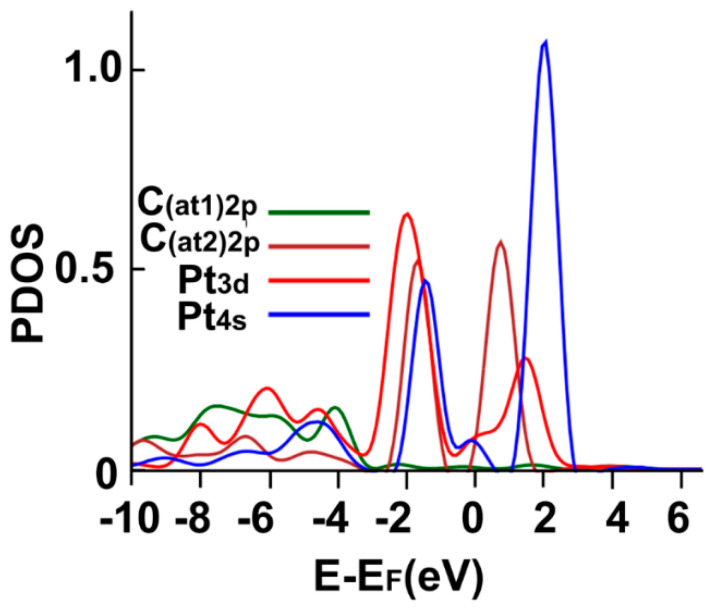
The PDOS for the adsorption of Pt on the graphene-C_30_ system for the pentagonal base.

Figure 7 shows the corresponding PDOS. We note the hybridization of orbital p from carbon with the orbitals s and p from platinum, around the Fermi energy, at around 2 eV above the Fermi energy, at about 2 eV below the Fermi energy, and below −4 eV.

### 2.6. Adsorption of Metals on the Graphene-C_30_ (H) Surface

#### 2.6.1. Doping with Li

**Figure 8:**
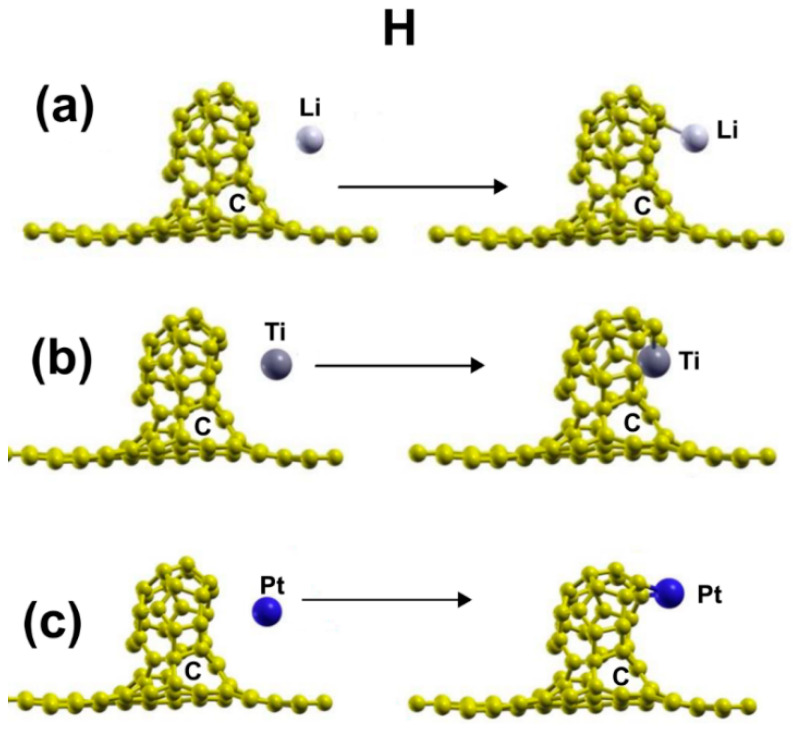
Adsorption of Li, Pt, and Ti on the graphene-C30 system for the hexagonal base. The three metals adsorbed strongly. (**a**) shows the initial and final configuration for the adsorption of a lithium atom on the surface. (**b**) shows the initial and final configuration for the adsorption of a titanium atom on the surface. (**c**) presents the initial and final configuration for the adsorption of a platinum atom on the surface.

Figure 8a shows the initial and final configuration for the adsorption of a lithium atom on the surface. The initial distance between the metal atom and the plane defined by the opening of C_30_ was 3.37 Å and 4.57 Å from the graphene layer. The lithium atom ends up bound to a carbon atom of C_30_ with an adsorption energy of −1.551 eV. It is a strong reaction but not as intense as the pentagonal case. The Li atom transfers 0.0364 electrons to the surface.

**Figure 9:**
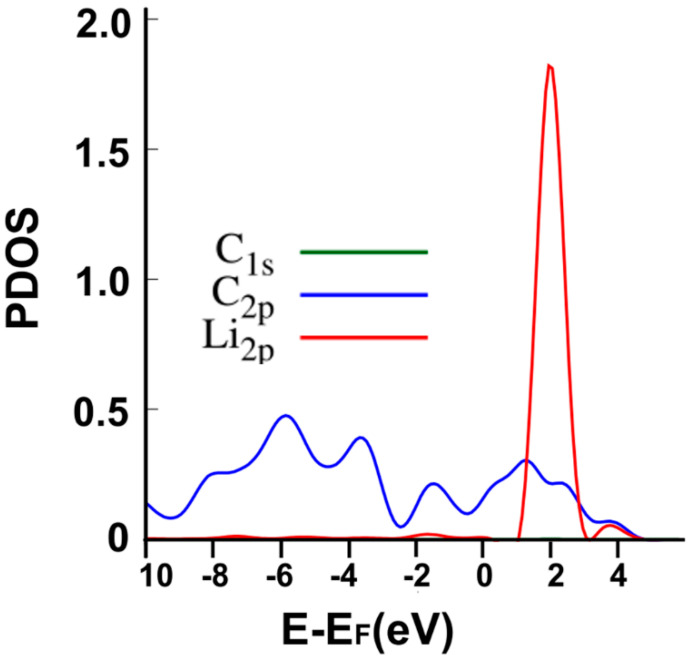
The PDOS for the adsorption of Li on the graphene-C_30_ system for the hexagonal base.

Figure 9 shows the interaction’s PDOS. Note the hybridization of orbitals s and p from carbon with the orbital p from lithium, between 1eV and 3 eV, around 4 eV, and a weaker hybridization between −2 eV and −1 eV.

#### 2.6.2. Doping with Ti

Figure 8b shows the initial and final configuration for the adsorption of a titanium atom on the surface. The initial distance between the metal atom and the plane defined by the opening of C_30_ was 3.34 Å and 4.57 Å from the graphene layer. The titanium atom ends up bound to two carbon atoms of C_30_. The adsorption energy of the titanium atom is −5.435 eV. The Ti atom transfers 0.6179 electrons to the system. The interaction is intense but not as much as in the pentagonal case.

**Figure 10:**
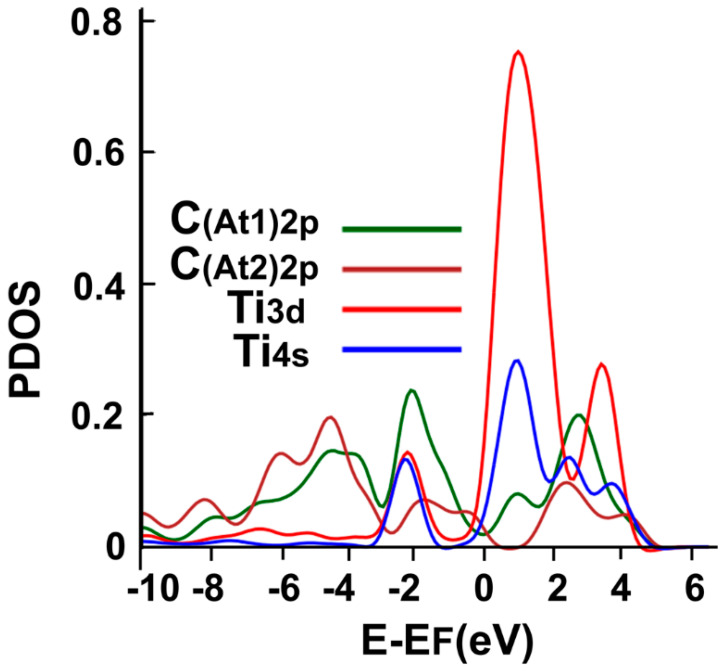
The PDOS for the adsorption of Ti on the graphene-C_30_ system for the hexagonal base.

We can see in Figure 10 the interaction’s PDOS. We note the hybridization of orbitals s and d from titanium with the orbitals p from the neighboring carbon atoms around −2 eV and between the Fermi energy and 5 eV.

#### 2.6.3. Doping with Pt

Figure 8c presents the initial and final configuration for the adsorption of a platinum atom on the surface. The initial distance between the metal atom and the plane defined by the opening of C_30_ was 3.34 Å and 4.57 Å from the graphene layer. The platinum atom ends up bound to two carbon atoms of C_30_. The adsorption energy of the platinum atom is −4.706 eV, which is a strong interaction with the surface but not as intense as in the pentagonal case.

**Figure 11:**
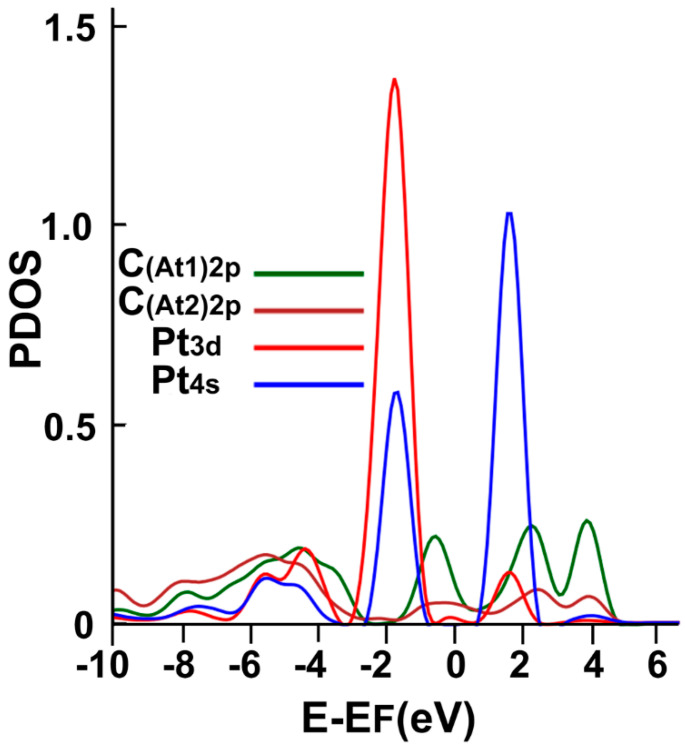
The PDOS for the adsorption of Pt on the graphene-C_30_ system for the hexagonal base.

The Pt atom transfers 0.5141 electrons to the surface. Figure 11 shows the corresponding PDOS. We Note the hybridization of orbital p from carbon atoms with the orbitals s and d from platinum, at around −2 eV, about 1.5 eV, and below −4 eV.

### 2.7. Adsorption of Pollutant Molecules on the Li-doped Graphene-C_30_ (P) Surface

#### 2.7.1. Adsorption of CO

There is no adsorption in this case.

#### 2.7.2. Adsorption of CO_2_

**Figure 12:**
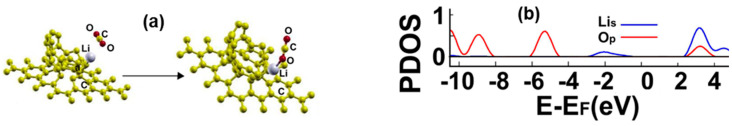
(**a**) The adsorption of CO_2_ on the Li-doped graphene-C_30_ system for the pentagonal base. The initial distance between the carbon atom of the CO_2_ molecule and the Li atom was 3.17 Å, and the distance from the graphene layer was 7.12 Å. The molecule was parallel to the graphene layer. The adsorption is without dissociation. (**b**) The PDOS for the adsorption of CO_2_ on the Li-doped graphene-C_30_ system for the pentagonal base.

Figure 12a shows the initial and final configuration of the system for the adsorption of a carbon dioxide molecule. The molecule ends up bound to the lithium atom via the oxygen atom with an adsorption energy of −0.373 eV. The molecule transfers 0.04688 electrons to the surface.

Figure 12b shows the corresponding PDOS. We note the hybridization of orbital p from the oxygen atom with the orbital s from lithium at around 3 eV.

#### 2.7.3. Adsorption of O_3_

**Figure 13:**
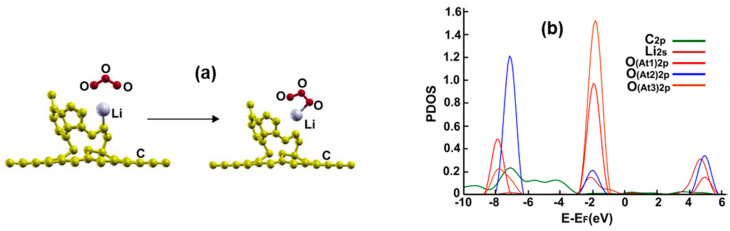
(**a**) The adsorption of O_3_ on the Li-doped graphene-C_30_ system for the pentagonal base. The initial distance between the central oxygen atom of the O_3_ molecule and the Li atom was 3.015 Å, and the distance from the graphene layer was 7.15 Å. The molecule was perpendicular to the graphene layer. The adsorption is without dissociation. (**b**) The PDOS for the adsorption of O_3_ on the Li-doped graphene-C_30_ system for the pentagonal base.

Figure 13a shows the initial and final configuration of the system for the adsorption of an ozone molecule. The molecule ends up bound to the lithium atom without dissociation. The adsorption energy is −1.777 eV, and using MD at 300 K, we found that the particle Li-O_3_ remains close to the surface at that temperature.

Figure 13b shows the corresponding PDOS. Notice the weak hybridization of orbitals p from the oxygen and carbon atoms with the orbitals s from the lithium between 0 and 2 eV.

### 2.8. Adsorption of Pollutant Molecules on the Ti-Doped Graphene-C_30_ (P) Surface

#### 2.8.1. Adsorption of CO

**Figure 14:**
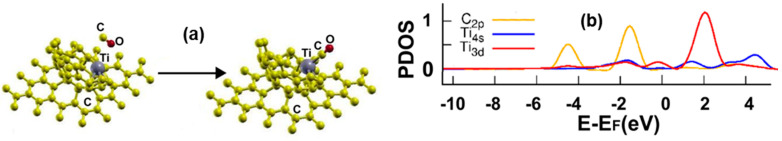
(**a**) Adsorption of CO on the Ti-doped graphene-C_30_ system for the pentagonal base. The initial distance between the carbon atom of the CO molecule and the Ti atom was 4.18 Å, and the distance from the graphene layer was 7.34 Å. The molecule was parallel to the graphene layer. The adsorption is without dissociation. (**b**) The PDOS for the adsorption of CO on the Ti-doped graphene-C_30_ system for the pentagonal base.

Figure 14a shows the initial and final configuration of the system for the adsorption of a carbon monoxide molecule. The molecule ends up bound to the titanium atom without dissociation via the carbon atom. The adsorption energy is −1.21 eV, and the molecule gains 0.0322 electrons from the surface.

Figure 14b shows the corresponding PDOS. Notice the hybridization of orbital p from the carbon atom with the orbitals s and d from the titanium atom at around −2 eV and between 1 eV and 4 eV.

#### 2.8.2. Adsorption of CO_2_

There is no adsorption in this case.

#### 2.8.3. Adsorption of CH_4_

There is no adsorption in this case.

#### 2.8.4. Adsorption of O_3_

**Figure 15:**
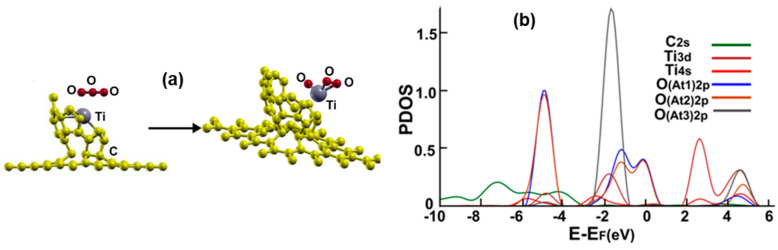
(**a**) Adsorption of O_3_ on the Ti-doped graphene-C_30_ system for the pentagonal base. The initial distance between the central oxygen atom and the Ti atom was 3.0 Å, and the distance from the graphene layer was 7.14 Å. The plane of the ozone molecule was parallel to the graphene layer. The adsorption is with dissociation. (**b**) The PDOS for the adsorption of O_3_ on the Ti-doped graphene-C_30_ system for the pentagonal base.

Figure 15a shows the initial and final configuration of the system for the adsorption of an ozone molecule. The molecule dissociates into an oxygen atom and an oxygen molecule. The oxygen atom is bound to the titanium, and the oxygen molecule is attached to the titanium atom. The adsorption energy of the ozone molecule is −6.3953 eV. The oxygen atom loses 0.2702 electrons. Besides, the oxygen molecule gains 0.4085 electrons. Using MD at 300 K, we obtained that the particle Ti-O_3_ remains close to the surface at that temperature.

Figure 15b shows the corresponding PDOS. Notice a weak hybridization of orbitals s from the carbon atom with the orbitals s and d from the titanium atom and p orbitals from the oxygen atoms at around 4 eV and between −6 eV and −4 eV with p orbitals from oxygen atoms and orbitals s from the titanium atom.

### 2.9. Adsorption of Pollutant Molecules on the Pt-Doped Graphene-C_30_ (P) Surface

#### 2.9.1. Adsorption of CO

There is no adsorption in this case.

#### 2.9.2. Adsorption of CO_2_

There is no adsorption in this case.

#### 2.9.3. Adsorption of CH_4_

There is no adsorption in this case.

#### 2.9.4. Adsorption of O_3_

**Figure 16:**
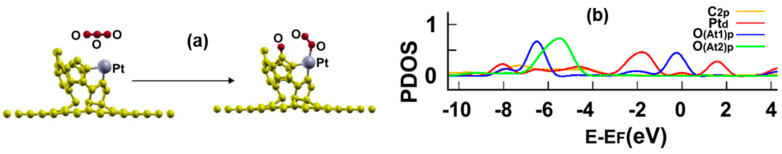
(**a**) Adsorption of O_3_ on the Pt-doped graphene-C_30_ system for the pentagonal base. The initial distance between the central oxygen atom and the Pt atom was 3.60 Å, and the distance from the graphene layer was 7.25 Å. The plane of the ozone molecule was parallel to the graphene layer. The adsorption is with dissociation. (**b**) The PDOS for the adsorption of O_3_ on the Pt-doped graphene-C_30_ system for the pentagonal base.

Figure 16a shows the initial and final configuration of the system for the adsorption of an ozone molecule. The adsorption energy is −0.8521 eV, and the molecule dissociates into an oxygen atom and an oxygen molecule. The oxygen atom ends up bound to a carbon atom. Besides, the oxygen molecule ends up bound to the platinum atom. The oxygen atom, which ends bound to a carbon atom, transfers 0.1207 electrons. The remaining part of the ozone molecule, the oxygen molecule bound to the Pt atom, gains 0.5665 electrons.

Figure 16b shows the corresponding PDOS. Notice a weak hybridization of orbitals p from the carbon atom with the orbitals p from the platinum and oxygen atoms at around 4.2 eV. The same hybridization is stronger below −4 eV.

### 2.10. Adsorption of Pollutant Molecules on the Li-Doped Graphene-C_30_ (H) Surface

#### 2.10.1. Adsorption of CO

There is no adsorption in this case.

#### 2.10.2. Adsorption of CO_2_

**Figure 17:**
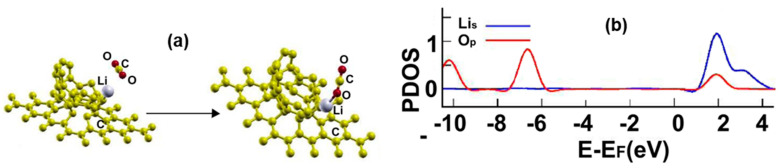
(**a**) Adsorption of CO_2_ on the Li-doped graphene-C_30_ system for the hexagonal base. The initial distance between the carbon atom of the CO_2_ molecule and the Li atom was 3.11 Å, and the distance from the graphene layer was 7.15 Å. The molecule was parallel to the graphene layer. The adsorption is without dissociation. (**b**) The PDOS for the adsorption of CO_2_ on the Li-doped graphene-C_30_ system for the hexagonal base.

Figure 17a shows the initial and final configuration of the system for the adsorption of a carbon dioxide molecule. The molecule adsorbs without dissociation, and one oxygen atom ends up bound to the lithium atom. The adsorption energy is −0.6491 eV, and the molecule transfers to the system 0.0803 electrons. The calculated recovery time at 300 K is 0.13 s, a good value for a sensor.

Figure 17b shows the corresponding PDOS. Notice the hybridization of orbitals p from the oxygen atom with the orbitals s from the lithium atom at around 2 eV.

#### 2.10.3. Adsorption of CH_4_

There is no adsorption in this case.

#### 2.10.4. Adsorption of O_3_

**Figure 18:**
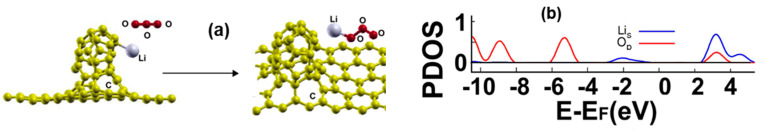
(**a**) Adsorption of O_3_ on the Li-doped graphene-C_30_ system for the hexagonal base. The initial distance between the central oxygen atom and the Li atom was 3.26 Å, and the distance from the graphene layer was 7.35 Å. The plane of the ozone molecule was parallel to the graphene layer. The adsorption is without dissociation. (**b**) The PDOS for the adsorption of O_3_ on the Li-doped graphene-C_30_ system for the hexagonal base.

Figure 18a shows the initial and final configuration of the system for the adsorption of an ozone molecule. The molecule ends up bound to the lithium atom without dissociation. The adsorption energy of the ozone molecule is −2.119 eV, and the surface transfers 0.2883 electrons to the ozone molecule. Using MD at 300 K, we found that the particle Li-O_3_ remains close to the surface at that temperature; it does not go away from the surface.

Figure 18b shows the corresponding PDOS. Notice the hybridization of orbitals p from the oxygen with the orbitals s from the lithium atom between 3 eV and 4 eV. There is a weaker hybridization below the Fermi energy.

### 2.11. Adsorption of Pollutant Molecules on the Ti-Doped Graphene-C_30_ (H) Surface

#### 2.11.1. Adsorption of CO

There is no adsorption in this case.

#### 2.11.2. Adsorption of CO_2_

There is no adsorption in this case.

#### 2.11.3. Adsorption of CH_4_

There is no adsorption in this case.

#### 2.11.4. Adsorption of O_3_

**Figure 19:**
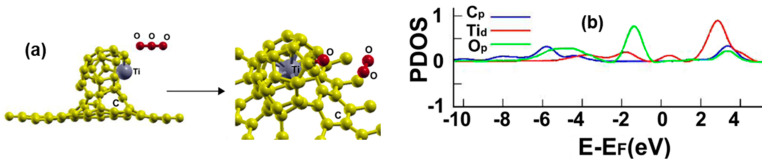
(**a**) Adsorption of O_3_ on the Ti-doped graphene-C_30_ system for the hexagonal base. The initial distance between the central oxygen atom and the Ti atom was 3.97 Å, and the distance from the graphene layer was 6.90 Å. The plane of the ozone molecule was parallel to the graphene layer. The adsorption is with dissociation into an oxygen atom and an oxygen molecule. (**b**) The PDOS for the adsorption of O_3_ on the Ti-doped graphene-C_30_ system for the hexagonal base.

Figure 19a shows the initial and final configuration of the system for the adsorption of an ozone molecule. The adsorption energy is −0.8214 eV, and the molecule dissociates into two fractions during adsorption, an oxygen atom and an oxygen molecule. Besides, the first fraction is bound to a carbon atom, and the second remains close to the surface. Using MD at 300 K, we found that the molecule O_2_ remains close to the surface at that temperature; it does not go away from the surface.

Figure 19b shows the corresponding PDOS. Notice the hybridization of orbitals p from the carbon and oxygen atoms and the orbitals d from the titanium atom between 2 and 4 eV and below the Fermi energy.

### 2.12. Adsorption of Pollutant Molecules on the Pt-Doped Graphene-C_30_ (H) Surface

#### 2.12.1. Adsorption of CO

**Figure 20:**
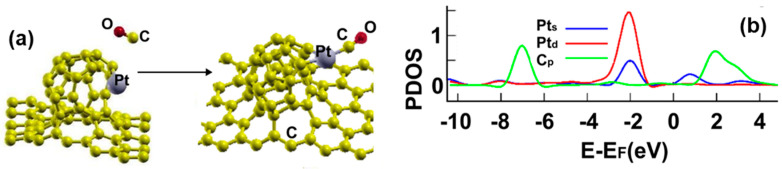
(**a**) Adsorption of CO on the Pt-doped graphene-C_30_ system for the hexagonal base. The initial distance between the center of the CO molecule and the Pt atom was 3.0 Å, and the distance from the graphene layer was 7.32 Å. The molecule was parallel to the graphene layer. The adsorption is without dissociation; (**b**) The PDOS for the adsorption of CO on the Pt-doped graphene-C_30_ system for the hexagonal base.

Figure 20a shows the initial and final configuration of the system for the adsorption of a carbon monoxide molecule. The adsorption energy is −1.756 eV without dissociation. The carbon atom ends up bound to the platinum atom.

The surface transfers 0.0322 electrons to the carbon monoxide molecule. Figure 20b shows the corresponding PDOS. We can see the hybridization of orbitals p from the carbon atom and the orbitals s from the platinum atom at around 3 eV and about 1.2 eV, respectively. We can also notice a weak hybridization of orbitals p from the carbon atom with orbitals d and s from the platinum atom below −1 eV.

#### 2.12.2. Adsorption of CO_2_

There is no adsorption in this case.

#### 2.12.3. Adsorption of CH_4_

There is no adsorption in this case.

#### 2.12.4. Adsorption of O_3_

**Figure 21:**
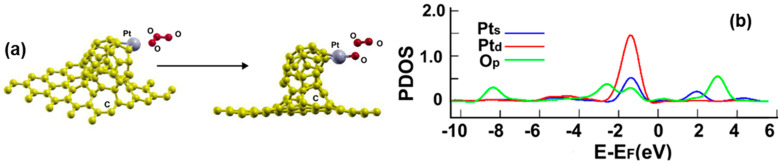
(**a**) Adsorption of O_3_ on the Pt-doped graphene-C_30_ system for the hexagonal base. The initial distance between the central oxygen atom and the Pt atom was 3.18 Å, and the distance from the graphene layer was 7.21 Å. The plane of the ozone molecule was parallel to the graphene layer. The adsorption is with dissociation. (**b**) The PDOS for the adsorption of O_3_ on the Pt-doped graphene-C_30_ system for the hexagonal base.

Figure 21a shows the initial and final configuration of the system for the adsorption of an ozone molecule that occurs with dissociation and with an adsorption energy of −1.43 eV. The molecule splits into two parts, an oxygen atom and an oxygen molecule. Using MD at 300 K, we found that the particle O_2_ remains close to the surface at that temperature; it does not go away from the surface.

The oxygen atom, which ends bound to the platinum atom, transfers 0.1207 electrons. The surface transfers 0.5665 electrons to the remaining fraction of the ozone molecule and the oxygen molecule, which remains close to the surface.

Figure 21b shows the corresponding PDOS. We can see the hybridization of orbitals p from the oxygen atom and the orbitals s and d from the platinum atom at around 2 eV and about −1.75 eV, respectively. We can also notice a weak hybridization of orbitals p from the oxygen atom with orbitals d and s from the platinum atom below −2 eV.

## 3. Materials and Methods

We used the GGA approximation for the exchange and correlation energies in the Perdew–Burke–Ernzerhohof (PBE) expression [26], using a Martins–Troullier norm-conserving pseudopotential [27]. We performed structural relaxations using the Quantum ESPRESSO code package [28], which uses periodical boundary conditions. We took threshold energy of 1.0 × 10^−6^ eV for convergence, a cut-off energy point of 1100 eV, and a threshold force of 1.0 × 10^−5^ eV/Å. We considered 40 k points within the Monkhorst–Pack particular k point scheme for Brillouin-zone integrations [29] with a separation of 0.083 Å^−1^.

**Figure 22:**
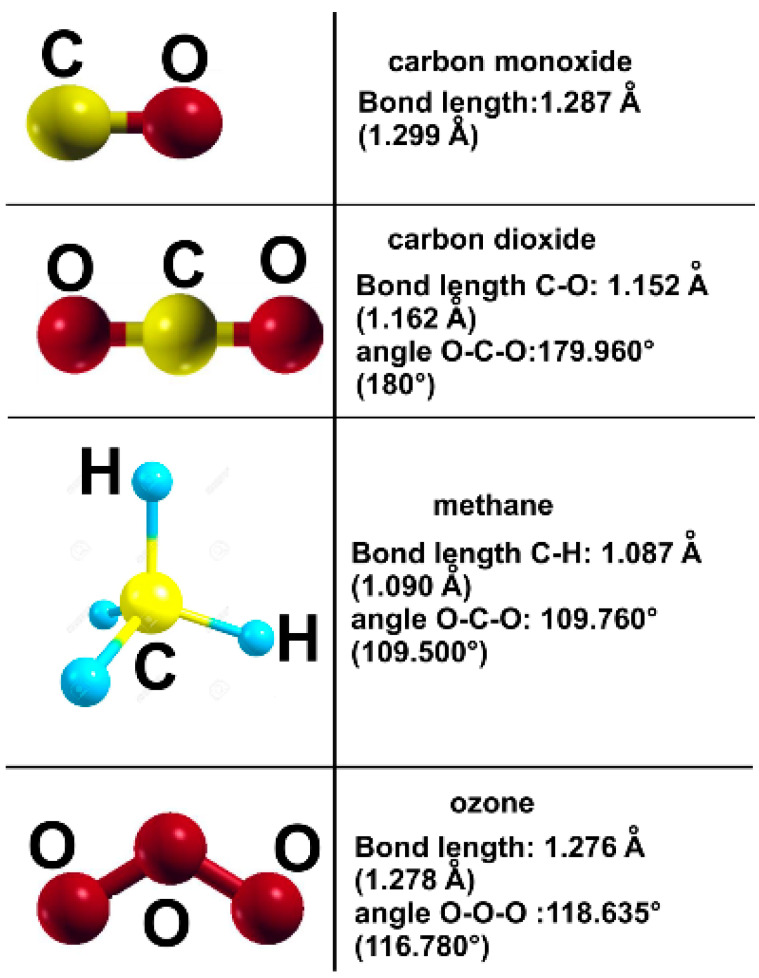
We compare our results, obtained by minimizing the molecule energy, and the experimental values are given in parenthesis.

To check the pseudopotentials, we minimized the energy of the different systems. Thus, we obtained the Li lattice parameter 3.495 Å (the experimental value is 3.510 Å) [30]; for titanium, we obtained: a = 2.863 Å, and c = 4.544 Å (the observed values are 2.950 and 4.683 Å, respectively [30]; in the case of Pt, we calculated a lattice parameter of 2.898 Å (the experimental value is 2.924 Å). We obtained the bond lengths and angles of the different pollutant molecules we are considering with the same approach. Figure 22 shows our results, which agree with the experimental values.

In our simulations, the adsorption energy is:

(1) $$E_{ads}=E\left(Surf+Mol\right)-\left[E\left(Surf\right)+E\left(Mol\right)\right],$$

where *E*(*Surf* + *Mol*) is the energy corresponding to the final system; [*E*(*Surf*) + *E*(*Mol*)] corresponds to the initial configuration, which is the energy of the surface, without interaction with the molecule plus the isolated molecule’s energy.

We calculated the recovery time (*τ*) from the Eyring transition state theory using the expression [31,32]:

(2) $$\tau =\left[h/\left(k_{B}\;T\right)\right]e^{-E_{ads}/\left(k_{B}T\right)}$$

In Equation (2), *h* is the Plank’s constant, *k_B_* is the Boltzmann’s constant, *E_ads_* is the adsorption energy, and *T* is the absolute temperature.

The desirable set of values for the recovery time is between 10^−2^ and ten seconds, implying at 300 K, adsorption energies in the range (−0.6428, −0.8215) eV.

## 4. Discussion

We performed computational simulations to investigate the adsorption of polluting molecules on graphene-semifullerene (C_30_) surfaces, considering two C_30_ geometries: hexagonal and pentagonal base. We found it possible to dope all surfaces with the metals Li, Ti, and Pt, which we used as catalysts in the adsorption of the different polluting molecules. We consider as pollutant molecules CO, CO_2_, CH_4_, and O_3_.

We obtained the semifullerene adsorbs on the graphene surface with adsorption energies of −14.97 eV and −16.41 eV, respectively, for pentagonal and hexagonal bases. The adsorption occurs on a six-vacancy cluster in a graphene layer. Besides, the catalysts adsorb on the graphene-C_30_ surface with a pentagonal base with adsorption energies of −4.02 eV, −6.3 eV, and −8.4 eV for Li, Pt, and Ti, respectively. For the hexagonal base, the adsorption energies are −1.87 eV, −4.7 eV, and −5.43 eV, in the same order. Notice that in each case (P or H), Li shows the adsorption energy with the minor magnitude and Ti with the largest.

The carbon monoxide molecule adsorbs on the pentagonal-base (P) surface only when Ti is the dopant, with an adsorption energy of −3.6 eV, and this adsorption is without dissociation. Furthermore, CO adsorbs on the hexagonal-base (H) surface only with Pt as the dopant with an adsorption energy of −0.89 eV. Again, the adsorption is without dissociation.

The carbon dioxide molecule adsorbs on both surfaces but only with Li as the dopant, with adsorption energies of: −0.67 eV for the P surface and −0.54 eV for the H surface. The adsorption of the CO_2_ molecule is without dissociation.

The methane molecule did not adsorb on any surface.

Finally, we found that both surfaces always adsorb the ozone molecule. When Ti or Pt are dopants, we found that the O_3_ molecule always dissociates into an oxygen molecule and an oxygen atom. For the P surface, the adsorption energies are −6.3953 and −0.8521 eV for the Ti and Pt doped surfaces, respectively. Furthermore, the adsorption energies for the Ti and Pt doped H surface are −0.82 eV and −1.43 eV, respectively. In the case of Li, the O_3_ molecule adsorbs without dissociation. The adsorption energy is −1.777 eV for the P surface, and the adsorption energy is −2.119 eV for the H surface.

At 300 K, the P surface would not act as a suitable sensor in any case. The H surface would be a sensor for O_3_ with Ti as the dopant (*τ* = 9.97 s) and for CO_2_ with Li as a dopant (*τ* = 0.13 s).

## Data Availability

Not applicable.
